# 非小细胞肺癌免疫检查点抑制剂治疗相关外周血生物标志物研究进展

**DOI:** 10.3779/j.issn.1009-3419.2021.102.24

**Published:** 2021-07-20

**Authors:** 鹏 王, 传昊 汤, 军 梁

**Affiliations:** 1 102206 北京，北京大学国际医院放疗科 Department of Radiation Oncology, Peking University International Hospital, Beijing 102206, China; 2 102206 北京，北京大学国际医院肿瘤内科 Department of Oncology, Peking University International Hospital, Beijing 102206, China

**Keywords:** 外周血标志物, 免疫检查点抑制剂, 肺癌, 免疫治疗, Blood biomarkers, Immmune Checkpoint Inhibitors, Lung neoplasms, Immunotherapy

## Abstract

以免疫检查点抑制剂（immune checkpoint inhibitors, ICI）为代表的免疫治疗改变了非小细胞肺癌（non-small cell lung cancer, NSCLC）的治疗模式，标志物指导下的免疫治疗是精准治疗的关键。基于组织的程序性死亡受体配体1（programmed cell death ligand 1, PD-L1）和肿瘤突变负荷（tumor mutational burden, TMB）是临床上广泛接受的用于指导免疫治疗的生物标志物，然而组织标本不易获取且难以克服肿瘤的时空异质性。外周血标志物作为组织检测的补充，具有取材方便、无创等优势，同时可涵盖肿瘤和宿主免疫状态两方面的信息，在NSCLC免疫治疗疗效预测及治疗反应动态监测方面的价值日益凸显。本文总结NSCLC免疫检查点抑制剂治疗相关外周血生物标志物的研究进展，旨在为开发新型的生物标志物提供参考。

## Abstract (en)

Immune checkpoint inhibitors (ICI) have transformed the treatment landscape of advanced non-small cell lung cancer (NSCLC). Biomarkers are essential for guiding precision immunotherapy. Tissue-based programmed death ligand 1 (PD-L1) expression and tumor mutational burden (TMB) are currently widely used biomarkers for selecting patients for immunotherapy. However, tissue specimens are often difficult to reach and couldn't overcome spatial and temporal heterogeneity. Blood biomarkers offer an alternative non-invasive solution that could provide a complete insight on patient's immune status and tumor as well, and show their potential in predicting the outcome as well as in monitoring response to immunotherapy. In this article, we summarize current knowledge on blood biomarkers in NSCLC patients treated with ICI, and we hope to provide more references for development of novel biomarkers.

近年来，以免疫检查点抑制剂（immune checkpoint inhibitors, ICI）为主的免疫治疗显著提高了非小细胞肺癌（non-small cell lung cancer, NSCLC）患者的客观缓解率（objective response rate, ORR）和总生存期（overall survival, OS），已经成为NSCLC治疗中不可或缺的部分。然而免疫治疗单药疗效有限，寻找特异的生物标志物，在治疗前筛选出免疫治疗优势人群是实现精准治疗的关键。目前多种基于组织的生物标志物已被证实可有效预测NSCLC免疫治疗疗效，主要包括：肿瘤组织程序性死亡受体配体1（programmed cell death ligand 1, PD-L1）表达水平、肿瘤突变负荷（tumor mutational burden, TMB）、微卫星不稳定（microsatellite instability, MSI）和错配修复基因缺陷（mismatch repair, MMR）、CD8^+^肿瘤浸润淋巴细胞（tumor infiltrating lymphocytes, TILs）、T细胞炎性基因表达谱和驱动基因[如表皮生长因子受体（epidermal growth factor receptor, *EGFR*）、间变性淋巴瘤激酶（anaplastic lymphoma kinase, *ALK*）、*STK11/LKB1*突变、*KRAS/TP53*共突变、鼠双微体2（murine double minute 2, *MDM2*）/*MDM4*扩增及*EGFR*扩增]等^[1]^。其中获得高级别证据支持的是肿瘤组织PD-L1表达水平及TMB。然而这些生物标志物尚未达到令人满意的临床预测效能。首先，部分PD-L1阴性或TMB低的患者也可能对免疫治疗产生应答；其次，基于小标本活检的检测结果无法克服肿瘤的时空异质性，难以反映肿瘤全貌及在病程中的动态变化；最后，基于组织的病理活检甚少反应宿主免疫状态，因而全身免疫状态的评估在肿瘤免疫治疗中的作用一直被低估。基于外周血的生物标志物可涵盖肿瘤和宿主微环境等多方面的信息且具有无创、实时、可反复取样等优势，在免疫治疗疗效预测及动态监测治疗反应方面显示出良好的应用前景。本文将对NSCLC免疫检测点抑制剂治疗相关外周血生物标志物的研究进展作一综述。

## 1 肿瘤相关的外周血标志物

### 1.1 外周血循环肿瘤细胞DNA（circulating tumor DNA, ctDNA）

ctDNA是指外周血中来源于肿瘤细胞的游离DNA，与肿瘤组织的DNA具有高度的一致性，基于ctDNA的无创活检在NSCLC的分子诊断、疗效监测等方面具有重要价值。目前基于ctDNA检测的NSCLC免疫治疗生物标志物的研究主要集中于两个方面：一是评估免疫治疗过程中ctDNA的动态变化与治疗反应及预后的关系；二是评估基于外周血ctDNA的TMB即blood TMB（bTMB）预测NSCLC免疫治疗疗效的可行性（表 1）。

**表 1:** 检测ctDNA和bTMB的不同研究的比较 Comparison of the different studies testing ctDNA and bTMB

Author, Year	Design	Population	Treatment	Method(s)/panel	Main findings/Threshold
Cabel, 2017^[2]^	*P*	NSCLC (*n*=10), UM (*n*=3), MSI-high CRC (*n*=2)	Nivolumab	ddPCR, bi-PAP, NGS monogene, 39 genes panel	ctDNA levels undetectable at 8 weeks associated with longer PFS and OS
Goldberg, 2018^[3]^	P	Metastatic NSCLC (*n*=28)	ICI, not further specified, CTx	NGS 24 genes panel	ctDNA diminution associated with prolonged survival; threshold ctDNA response as a > 50% decrease in AF from baseline
Giroux, 2018^[4]^	P	Stage Ⅲb/Ⅳ NSCLC (*n*=15)	Nivolumab	NGS 22 genes panel	9% increase of ctDNA at first tumor evaluation correlated with absence of clinical benefit, shorter PFS and poorer OS
Passiglia, 2019^[5]^	R	Stage Ⅳ NSCLC (*n*=45)	Nivolumab	qPCR unknow	cfDNA increase > 20% at 6 weeks associated with worse OS and shorter TTP
Guibert, 2019^[6]^	R	Stage Ⅲb/Ⅳ, progressive NSCLC (*n*=65)	ICI	NGS 36 genes panel	Early changes (increase *vs* decrease) in the ctDNA AF were correlated with PFS
Anagnostou, 2019^[7]^	R	Metastatic NSCLC (*n*=24)	ICI	TEC-Seq NGS 58 genes panel	Reduction in ctDNA to undetectable levels was associated with longer PFS and OS
		Stage Ⅰ-Ⅲ NSCLC (*n*=14)	Neo-adjuvant nivolumab		Reduction in ctDNA to undetectable levels was associated with major or partial pathological response
Zhang, 2020^[8]^	R	Advanced lung cancer (*n*=333) Other solid tumors (*n*=645)	Durvalumab±tremelimumab	NGS 73 genes panel	Pretreatment VAF was inverse correlated with OS; ctDNA increased during treatment was correlated with poor OS
Gandara, 2018^[10]^	R	Advanced NSCLC: OAK (*n*=273) +POPLAR (*n*=583)	Atezolizumab	F1CDx 324 genes	bTMB is positively associated with PFS threshold 16 Mu*t*/mb
MYSTIC, 2020^[11]^	R	Metastatic NSCLC: MYSTIC (*n*=809)	Durvalumab+ tremelimumab	GuardantOMNI 500 genes	bTMB is positively associated superior ORR, PFS and OS; threshold 20 Mu*t*/mb
Wang, 2019^[12]^	R	Advanced NSCLC (Line 1: *n*=48; Line 2: *n*=50)	ICI, non-specified	NCC-GP150 150 genes	bTMB is positively associated with superior ORR and PFS; threshold 6 Mu*t*/mb
Nabet, 2020^[13]^	R	Advanced NSCLC (*n*=99)	ICI	CAPP-Seq 270 genes	High blood based TMB, ctDNA decreased after one infusion, low CD8 are associated with good DCB; threshold 14 Mu*t*/mb
R/P: retrospective/prospective design; NSCLC: non-small cell lung cancer; UM: uveal melanoma; MSI: microsatellite instability; CRC: colorectal cancer; ddPCR: droplet-digital polymerase chain reaction; bi-PAP: bidirectional pyrophosphorolysis activated polymerization; NGS: next generation sequencing; PFS: progression free survival; OS: overall survival; ICI: immune checkpoint inhibitor; CTx: chemotherapy; AF: allele fraction; qPCR: quantitative polymerase chain reaction; TTP: time to progression; TEC-Seq: targeted error corrected sequencing; VAF: variant allele fraction; bTMB: blood tumor mutational burden; CAPP-Seq: cancer personalized profiling by deep sequencing; DCB: durable clinical benefits; ctDNA: circulating tumor DNA; bTMB: blood tumor mutational burden.					

多项研究^[2-8]^中观察到免疫治疗过程中ctDNA水平下降与患者疾病缓解及生存提高相关，且从治疗开始至观察到ctDNA应答的时间要明显早于观察到影像学变化的时间（24.5 d *vs* 72.5 d）^[3]^，提示通过检测外周血ctDNA的变化可以较影像学更早的识别肿瘤对免疫治疗的反应性，有利于指导临床决策。然而并不是所有患者都可以在基线评估时检测到ctDNA，在恶性黑色素瘤相关研究中，有文献^[9]^报道出现中枢神经系统疾病进展的患者在基线和随后的评估中均检测不到ctDNA，其可能与血脑屏障有关，NSCLC同样具有较高的脑转移发生率，是否也面临类似的问题需要进一步研究证实。

TMB代表肿瘤基因组编码区内的体细胞突变数量，目前认为TMB越高，肿瘤免疫原性越强，越容易被T细胞识别，对ICI应答反应越好。与PD-L1一样，TMB也面临着肿瘤时空异质性的问题，能否以bTMB代替组织TMB（tissue TMB, tTMB）预测免疫治疗疗效成为目前研究的热点。多项研究显示在NSCLC中bTMB与tTMB具有显著相关性^[10-12]^，且高bTMB与接受ICI治疗获益显著相关^[10-13]^，Gandara等^[10]^回顾性分析来自POPLAR和OAK两个队列中的数据，发现在Atezolizumab治疗前具有高bTMB水平（≥16个突变/Mb）的NSCLC患者有更好的无进展生存期（progression-free survival, PFS），国内的一项回顾性研究^[12]^亦显示高bTMB（≥6个突变/Mb）组患者的PFS和ORR较低bTMB（< 6个突变/Mb）组显著改善，而随后的MISTIC研究^[11]^观察到高bTMB（≥20个突变/Mb）的患者不仅有更好的ORR、PFS，也显示出显著的OS获益。目前全外显子测序（whole exon sequencing, WES）是评估组织TMB的“金标准”，但因成本高、样本需求量较大、数据分析较复杂，难以常规应用于临床，通过靶向基因测序panel（next generation secquencing panel, NGS panel）检测TMB是可行的替代手段，值得注意的是，靶向测序panel的大小是影响TMB评估置信区间及阈值的重要参数，这也是上述采用不同测序平台的各系列研究所得的bTMB阈值不同的一个重要原因，同时除在MYSTIC小样本研究结果中观察到高bTMB患者有显著的OS获益外，其他研究中所得的bTMB cut-off值均无法区分ICI治疗后OS显著获益的人群。新近发布的一项前瞻性研究（B-F1RST）^[14]^评估bTMB对Atezolizumab一线治疗晚期NSCLC疗效预测的作用，结果显示bTMB≥16个突变/Mb组患者的PFS和OS较bTMB < 16个突变/Mb组有绝对值的明显延长（中位PFS：5.0个月*vs* 3.5个月；中位OS：23.9个月*vs* 13.4个月），然而也同样未达到统计学差异。目前认为bTMB对OS预测效能不足与其基于血液的采样方法相关，bTMB的检测依赖于肿瘤释放的ctDNA含量，研究^[15]^证实bTMB水平与ctDNA含量呈正相关，而ctDNA含量高提示肿瘤负荷大，与不良预后相关，因此高ctDNA对预后的负向预测作用会部分抵消高bTMB对免疫治疗疗效的正向预测作用，通过优化bTMB的计算方法可改善其预测效能^[13, 15]^。国外有研究^[13]^拟合ctDNA与bTMB得到normalized bTMB，发现与bTMB相比，normalized bTMB与ICI治疗获益的相关性更为显著。而国内的研究发现bTMB中的高频等位基因突变（allele fraction, AF）更能反映肿瘤负荷，而超低频等位基因突变在一定程度反映肿瘤的异质性，二者均与不良预后相关，通过剔除高频突变（AF > 5%）^[15]^或超低频突变[AF/最大体细胞等位基因突变频率（maximum somatic allele frequency, MSAF） < 10%]^[16]^得到的优化bTMB较传统bTMB具有更高的预测效能，以上两项研究应用已发表的POPLAR和OAK两个队列的数据对矫正后的bTMB的预测效能进行验证，均得到了能在OS上显著区分免疫治疗获益人群的cut-off值^[15, 16]^，当同时剔除高频突变及超低频突变，bTMB的预测效能进一步提升^[16]^。克隆性造血可能会影响ctDNA检测的准确性进而影响bTMB的计算，可以通过优化ctDNA检测的基因-panel来规避其带来的影响，国内已有研究^[12]^验证了基于NCC-GP150 panel计算的TMB与全外显子计算的TMB有很好的相关性，此外，优化bTMB的算法以及建立克隆性造血的突变数据库也是可选择的提高bTMB准确性的方法。与tTMB相比，bTMB可重复检测并能克服肿瘤的时空异质性，在NSCLC免疫治疗疗效预测方面已经积累了一定的临床证据，如何进一步优化bTMB，达到预测效能和涵盖人群的有效平衡将是未来研究的重点。

### 1.2 循环肿瘤细胞

循环肿瘤细胞（circulating tumor cells, CTCs）检测在肺癌治疗耐药性监测及预后判断方面具有重要价值，但其在肺癌免疫治疗中应用的价值尚不明确。Nicolazzo等^[17]^检测24例Ⅳ期NSCLC患者ICI治疗前后外周血中CTCs数目及CTCs上PD-L1的表达情况，发现在治疗后6个月，所有外周血中检测到PD-L1阳性CTCs的患者均出现了疾病进展，提示CTCs上PD-L1的表达情况可能具有提示预后的作用。Guibert等^[18]^检测96例患者Nivolumab治疗前CTCs上PD-L1表达情况，发现PD-L1^+^ CTCs计数 > 1%的患者治疗无响应的比例更高，Janning等^[19]^的研究发现ICI治疗后病情进展的患者均出现PD-L1^+^ CTCs计数增加，而治疗获益患者PD-L1^+^ CTCs计数不变或下降，上述两项研究^[18, 19]^均未观察到CTCs上PD-L1的表达与组织PD-L1表达存在相关性。目前已发表的少数研究^[18-20]^尚不能证明CTCs上PD-L1的表达情况与NSCLC免疫治疗疗效存在确切的相关性，加之CTCs检测花费高、耗时等缺点更限制了其在临床上的广泛应用。

### 1.3 外泌体

肿瘤细胞分泌的外泌体带有与原始细胞更为接近的蛋白组分及遗传信息（DNA、RNA），是外泌体作为组织检测补充的理论基础。肿瘤细胞释放的外泌体可表达PD-L1（exosomal PD-L1, ePD-L1），Del等^[21]^在小样本黑色素瘤和NSCLC患者免疫治疗群体中观察到，ePD-L1 mRNA水平与治疗反应显著相关。然而PD-L1 mRNA表达受多方面因素调控，因此国内有研究^[22]^探讨直接检测ePD-L1蛋白的可行性，结果显示肺癌及乳腺癌细胞系PD-L1表达及其分泌外泌体ePD-L1的表达存在一定的差异；与之类似，Li等^[23]^的研究发现NSCLC患者ePD-L1水平与肿瘤大小、分期正相关，但与对应肿瘤组织的PD-L1表达无显著相关性，ePD-L1水平是否能反映原肿瘤组织的PD-L1表达水平仍需进一步研究证实。ePD-L1与NSCLC免疫治疗预后的关系尚未见相关报道，一项评估ICI联合化疗一线治疗晚期NSCLC患者外泌体ePD-L1及miRNA表达谱与预后关系的临床试验（NCT04427475）正在招募中。

外泌体miRNA可通过参与免疫抑制和调节PD-L1表达等多种途径影响免疫系统功能，彭等^[24]^的研究比较接受ICI治疗的NSCLC患者与健康对照个体血浆外泌体miRNA表达谱的差异，结果显示疾病进展组患者基线miR-320家族miRNA较疾病缓解组患者和健康个体明显上调；疾病缓解组患者免疫治疗后miR-125b-5p较基线明显下调，提示miR320家族和miR-125b-5p或可作为NSCLC免疫治疗疗效预测的生物标志物。

### 1.4 血浆可溶性蛋白

外周血PD-L1除上述提到的CTCs上的PD-L1和ePD-L1，还有血浆可溶性PD-L1（soluble PD-L1, sPD-L1），sPD-L1同样可以与T细胞表面程序性死亡受体-1（programmed cell death 1, PD-1）结合，导致T细胞失活，负向调控免疫系统。Okuma等^[25]^分析39例NSCLC患者Nivolumab治疗前血浆中sPD-L1水平与预后的关系，发现基线sPD-L1水平低的患者有更高的疾病缓解率和更长的OS。Costantini等^[26]^监测NSCLC患者治疗过程中sPD-L1动态变化，发现Nivolumab治疗后2个月治疗应答组患者血浆中sPD-L1水平显著高于无应答组。目前对sPD-L1的定量检测主要是通过酶联免疫吸附测定（enzyme linked immunosorbent assay, ELISA）法，然而该种检测技术灵敏度和重复性较差，sPD-L1的最佳检测技术及用于疗效预测判定的阈值仍需进一步的研究确定。

## 2 宿主相关外周血标志物

### 2.1 外周血免疫细胞

免疫细胞在机体抗肿瘤免疫中发挥至关重要的作用，免疫细胞的类型、数量及功能决定了机体抗肿瘤免疫的效果。因此监测外周血免疫细胞的变化可反映机体的免疫状态及对免疫治疗的反应性，有望成为预测免疫治疗疗效的生物标志物（表 2）。

**表 2:** 外周血免疫细胞与非小细胞肺癌免疫治疗反应的关系 Levels of peripheral immune cells correlated with immunotherapy response in NSCLC

Cell type	Biomarkers	Clinical benefit	Reference
Blood routine examination	Baseline ANC < 7, 500/μL, ALC≥1, 000/μL, AEC≥150/μL, NLR < 5	OS, PFS	[27-29]
Cells	Baseline NLR < 6.4, PLR < 441.8, dNLR≤3	OS	[31, 30]
	Post-treatment NLR < 5 at week 6	OS, PFS	[32]
CD8^+^ T cells	High baseline expression of immune checkpoints (PD-1)	OS, PFS	[33]
	Low baseline expression of PD-1		
	Decreased expression of PD-1 after treatment	OS, PFS	[35]
	Without increased expression of immune checkpoints (TIM-3^+^) after treatment	PFS	[36]
	High proliferation of PD-1+CD8^+^ T cells after anti-PD-1 therapy	PR/SD, DCB, PFS	[37, 38]
	High baseline TCR diversity in PD-1^+^CD8^+^ T cells	PFS	[39]
	Increased TCR diversity in T cells（including CD8^+^ T）at 2 weeks after treatment	OS	[40]
	Low baseline frequency of CD28^-^CD57^+^KLRG1^+^	OS	[42]
	Expression of CD28 and ICOS after anti-PD-1 therapy	PR/SD	[37]
	Lack CD28, ICOS and CD40L	PR/SD	[44]
	Higher baseline memory CD8^+^ T cells (CM/Eff T cell ratio)	PFS	[45]
CD4^+^ T cells	High baseline expression of immune checkpoints (PD-1)	PFS	[46]
	Higher baseline frequency of functional CD27^-^CD28^-^CD4^+^ T cells	PFS	[47]
	High frequencies of Treg cells one week after anti-PD-1 therapy	OS, PFS	[48]
NK cells	Higher frequency and overall activity of NK cells	PR, SD	[49]
	High baseline number of NK cells	OS, PFS	[33]
	Low baseline number of NK cells	OS, PFS	[34]
MDSCs	Low baseline frequency of PMN-MDSCs and M-MDSCs	OS, PFS	[50]
	Low numbers of M-MDSC 2 weeks after nivolumab therapy	OS	[51]
	High baseline levels of Gr-MDSC	OS, PFS	[52]
Combination cells	Higher baseline (%CD62L^low^CD4^+^ T cells)^2^/(%Treg cells) ratio PFS and OS	OS, PFS	[53]
	Higher (%Treg cells)/(%LOX-1^+^ PMN-MDSCs) ratio after the first nivolumab infusion	PFS	[54]
	(%NK cells)/(%Lox1^+^ PMN-MDSC) ratio≥5.75）after the first cycle of anti-PD-1 therapy	ORR, OS, PFS	[55]
ALC: absolute lymphocyte count; ANC: absolute neutrophil count; AEC: absolute eosinophil count, NLR: neutrophil-to-lymphocyte ratio; dNLR: derive neutrophil-to-lymphocyte ratio; PLR: platelet-to-lymphocyte ratio; PD-1: programmed death-1; TIM-3: T-cell immunoglobulin mucin 3; PR: partial response; SD: stable disease; cm/Eff: central memory/effector memory; TCR: T cell receptor; ICOS: inducible co-stimulator; M-MDSC: monocytic-myeloid-derived suppressor cell; Gr-MDSCs/PMN-MDSCs: granulocytic/polymorphonucear-myeloid-derived suppressor cells.			

#### 2.1.1 基于血常规的预测标志物

外周血中淋巴细胞、粒细胞等的变化可在一定程度上反映机体的免疫状态并具有一定的疗效预测作用，研究^[27]^发现NSCLC患者基线嗜酸性粒细胞计数高、淋巴细胞计数高及中性粒细胞计数低与ICI治疗后更好的OS相关，此外由血常规分类细胞计数衍生的系统性炎性指标如中性粒细胞淋巴细胞比值（neutrophil-to-lymphocyte ratio, NLR）、衍生NLR（derive NLR, d NLR）、血小板淋巴细胞比值（platelet-to-lymphocyte ratio, PLR）与ICI治疗疗效也具有一定的相关性，NSCLC患者ICI治疗前高NLR^[28, 29]^、dNLR^[30]^、PLR^[31]^及治疗早期高NLR水平^[32]^与更差的OS和PFS相关。血常规容易获取，但也容易受到多种因素干扰，因此基于血常规的预测标志物仅能辅助或联合其他指标共同判断预后。

#### 2.1.2 CD8^+^ T细胞

CD8^+^ T淋巴细胞介导的细胞免疫是机体抗肿瘤免疫应答的基础，CD8^+^ T细胞的活化或抑制需要双信号的刺激，第一信号为T细胞受体（T cell receptor, TCR）识别肿瘤抗原，第二信号为B7-CD28家族（共刺激受体或共抑制受体）识别协同刺激分子。肿瘤状态下，CD8^+^ T细胞处于耗竭、衰老或失能的免疫抑制状态，从而失去对肿瘤的免疫监视功能。ICI主要是通过阻断T细胞上共抑制受体如PD-1、细胞毒性T淋巴细胞相关蛋白4（cytotoxic T-lymphocyte-associated protein 4, CTLA-4）等介导的免疫抑制通路，重新激活T淋巴细胞、逆转CD8^+^ T细胞的耗竭状态发挥抗肿瘤作用。目前认为持续过表达共抑制受体如PD-1、CTLA-4和T细胞免疫球蛋白黏蛋白3（T-cell immunoglobulin mucin 3, TIM-3）等为耗竭T细胞的表型标志，多项研究^[33-36]^表明NSCLC患者外周血CD8^+^ T细胞中耗竭T细胞亚群占比与ICI治疗疗效相关，如有研究^[33]^报道基线PD-1^+^CD8^+^ T细胞水平高的NSCLC患者接受Nivolumab后可获得更长的OS，然而也有研究^[34]^中观察到基线PD-1^+^CD8^+^ T细胞水平低的患者更易对Nivolumab产生应答反应；在ICI治疗过程中，外周血CD8^+^ T细胞PD-1^+^表达持续下降且下降幅度超过2%的患者可获得更优的PFS及OS^[35]^，而TIM-3^+^表达升高的患者表现出更差的PFS^[36]^，此外ICI治疗后PD-1^+^CD8^+^ T细胞出现活跃增殖反应的患者更易对ICI产生应答反应^[37, 38]^并获得更长的PFS^[38]^。TCR是T细胞识别肿瘤抗原的效应器，有研究^[39]^发现基线外周血PD-1^+^CD8^+^ TCR多样性水平高和ICI治疗后出现PD-1^+^CD8^+^ TCR克隆增殖的患者PFS明显延长，新近亦有研究^[40]^报道，Durvalumab治疗后患者外周血T细胞TCR多样性增加的患者可获得更长的OS。

共刺激受体CD28与B7分子的结合是T细胞活化最为基本的协同刺激信号，CD28下调是CD8^+^ T细胞衰老的表型标志，与对ICI治疗抗拒有关^[41]^，研究^[42]^发现基线为T细胞衰老免疫表型（senescent immune phenotype, SIP）（即外周血CD8^+^ T细胞中CD28^-^CD57^+^KLRG1^+^细胞占比高）与NSCLC患者接受ICI后更差的PFS和OS相关。诱导协同刺激分子（inducible co-stimulator, ICOS）同属CD28家族，仅在T细胞活化后被诱导表达，ICOS既可以通过增强CD8^+^ T的效应功能发挥抗肿瘤作用，也可以通过调控调节性T细胞（regulatory cells, Treg）细胞发挥免疫抑制作用，在不同肿瘤中与预后的关系也不尽相同^[43]^。在小样本NSCLC研究中发现80%对ICI产生应答反应的患者可以在ICI治疗后检测到PD-1^+^CD8^+^ T细胞的活跃增殖，且这部分细胞共表达CD28和ICOS^[37]^，然而也有研究报道NSCLC患者对Nivolumab的应答反应与基线时CD45RA^+^CCR7^−^CD8^+^ T细胞缺乏共刺激受体CD28、ICOS和CD40L相关^[44]^。

记忆性T细胞在肿瘤免疫应答和免疫记忆维持中发挥重要作用。记忆性T细胞分为中心记忆性T细胞（central memory T cell, cm）和效应记忆性T细胞（effector memory T cell, Eff）。cm细胞具有长期记忆性，Eff细胞是被激活的cm细胞，有研究报道Nivolumab治疗前外周血cm/Eff CD8^+^ T细胞高的患者PFS明显延长^[45]^。

#### 2.1.3 CD4^+^ T细胞

CD4^+^ T细胞是调控免疫反应最重要的枢纽细胞，研究^[46]^发现基线PD-1^+^CD4^+^ T细胞水平高与NSCLC患者接受ICI治疗后更长的PFS相关。CD27^-^CD28^-^ T细胞被认为是具有记忆和效应功能的高度分化的T细胞，有研究^[47]^显示ICI治疗前外周血CD4^+^ T细胞中CD27^-^CD28^-^ T占比高的患者有更好的PFS，提示辅助性CD4^+^ T细胞在ICI治疗中同样发挥着不可或缺的作用。Treg细胞主要为CD4^+^CD25^+^FoxP3^+^ T细胞，是维持机体免疫耐受的重要因素之一，有研究^[48]^发现免疫治疗后外周血Treg细胞水平高的NSCLC患者有更好的应答反应和总生存。

#### 2.1.4 其他免疫细胞

自然杀伤（natural killer, NK）细胞是机体清除肿瘤细胞的第一道防线，在NSCLC中有研究^[33, 49]^显示免疫治疗前外周血NK细胞水平高与更好的治疗反应及预后相关，然而也有研究^[34]^得出相反的结论。骨髓源性抑制性细胞（myeloid-derived suppressor cells，MDSCs）是一组具有免疫抑制潜能的异质细胞群，关于MDSCs与NSCLC免疫治疗疗效的关系的研究较少且各研究^[50-52]^报道结果不一。

外周血各免疫细胞亚群的占比、表型等可反映宿主的免疫状态及对免疫治疗的反应性，显示出一定的疗效预测潜能，然而免疫细胞种类繁杂，彼此间相互作用，单一的免疫细胞标志物的预测作用可能有限，已有学者联合多种免疫细胞指标预测免疫治疗疗效^[53-55]^，目前以外周血免疫细胞作为ICI治疗疗效预测标志物的研究多处于临床前研究阶段，寻找特异性的检测指标，确定检测的最佳时机及各指标的判定阈值都需要更为深入的研究（表 3）。

**表 3:** 免疫检查点抑制剂治疗相关外周血标志物的优缺点 Advantages and limitations of the main blood biomarkers under investigation in the area of immune checkpoint inhibitors-based therapy

Item	Composition	Advantages	Disadvantages	Level of evidence
ctDNA levels	Nucleic acid	Highly specific and sensitive, real time quantitative analysis enable dynamic evaluation of tumor at a precise moment, covering spatial and temporal tumor heterogeneity	Lack of standardization of pre-analytical and detection methods, time-consuming	Prospective study
bTMB	Nucleic acid	Standardized detection technology: WES is the gold standard while NGS can serve as a sufficiently fast candidate tests, covering spatial and temporal tumor heterogeneity	Lack of standardization of pre-analytical methods. WES: long and very expensive, NGS: optimal gene panel size, algorithm and a consensual cut-off defining high TMB are still to be determined, expensive	Prospective study
CTC	Living cells	Specific, single-cell analysis. CellSearch: standardized, semiautomated, covering spatial and temporal tumor heterogeneity	Very rare, hard to keep, variability of technologies, expensive	Retrospective study
Exosomes	Nucleic acid, protein	Widely distributed and good stability, unique surface protein and genetic material originated from their parental cells, covering spatial and temporal tumor heterogeneity	Technology for exosomal isolation and tests is not broadly available	Retrospective study
Circulating immune cells	Immune cell subpopulations	Reflecting the host's immune status, Simultaneous detection of multiple subpopulations	Lack of standardized methodological approaches, complex classification, highly dynamic and the optimal target and detecting timing are still to be determined, long technical and analysis time	Retrospective study
WES: whole exon sequencing; CTCs: circulating tumor cells.				

### 2.2 可溶性全身免疫和炎症标志物

颗粒酶B（granzyme B）是由NK细胞和细胞毒性CD8^+^ T分泌的发挥细胞免疫功能的重要效应蛋白，研究^[26]^发现Nivolumab治疗前血清中颗粒酶B浓度低的NSCLC患者有更好的治疗反应性及更优的生存。吲哚胺2, 3-二加氧酶（indoleamine 2, 3-dioxygenase, IDO）通过降解T细胞增殖所需的必需氨基酸色氨酸发挥免疫抑制作用，IDO活性的增加与对ICI治疗抗拒相关，小样本临床研究结果^[56]^显示晚期NSCLC患者二线免疫治疗中，IDO活性与PFS和OS呈负相关。乳酸脱氢酶（lactate dehydrogenase, LDH）及C-反应蛋白（C-reactive protein, CRP是临床上常用的非特异的指标，回顾性研究结果显示免疫治疗前LDH^[57]^及CRP^[58]^升高的NSCLC患者预后更差，同时在前瞻性研究中也观察到Atezolizumab治疗后CRP下降的患者有着更好的OS和PFS^[14]^。其他炎症标志物如炎性因子与ICI治疗疗效的关系也有少量文献报道，如治疗过程中IL-8水平下降^[59]^以及肿瘤坏死因子-α（tumor necrosis factor-α, TNF-α）、γ-干扰素（interferon γ, IFN-γ）水平升高^[60]^的NSCLC患者可能更容易从ICI治疗中获益。全身免疫和炎症标志物同样面临容易受干扰、特异性差的问题，仅能在部分经选择的人群中作为辅助的标志物。

## 3 多变量预测模型标志物

将涵盖肿瘤和宿主信息的多个生物标志物联合作为一个复合变量可提高预测的特异性。如有研究^[61]^报道ICI治疗前外周血检测结果为高sPD-L1、低CD8^+^PD-1^+^ T细胞且低NK细胞的患者表现出更差的OS和PFS。新近的一项研究^[13]^应用贝叶斯框架整合不同的风险预测因子，构建ICI治疗疗效预测的生物标志物模型并进行验证，其中的DIREct-On模型纳入治疗前外周血CD8^+^ T细胞水平和bTMB以及治疗过程中ctDNA动态改变，结果显示DIREct-On分数高的患者可获得更长的PFS，且该预测模型对疗效预测的准确性高于任意单个预测因子。这些研究表明开发预测效能更高的多变量生物标志物可能是未来的研究方向之一。

## 4 免疫相关不良反应（immune-related adverse reactions, irAE）标志物

ICI的免疫活化作用降低了人体的免疫耐受性，在增强机体抗肿瘤反应的同时，也导致了irAEs的发生，70%的患者在使用ICI过程中可发生不同等级的irAEs，寻找irAEs预测生物标志物，对改善患者预后具有重大意义。研究发现免疫治疗前外周血NLR及PLR水平低与更高的irAEs发生率相关^[62]^，同时NLR的变化与irAEs的临床病程相关，在最早出现症状并诊断间质性肺炎的4周前即可出现NLR的明显升高，且在严重的irAEs事件后NLR呈明显回落的患者表现出更好的临床转归及更长的PFS^[63]^。此外，有研究^[26]^报道基线IL-2水平低及治疗后2个月IFN-γ水平高可能与Nivolumab治疗后更高的3级-4级不良反应发生率相关。目前关于irAEs相关外周血标志物的研究较少，寻找有意义的irAEs的生物标志物是今后免疫治疗领域面临的又一巨大挑战。

## 5 总结与展望

外周血生物标志物具有无创、可重复检测等优势，不仅可以克服肿瘤的时空异质性，还可以监测治疗过程中肿瘤生物学及机体免疫状态的动态变化，具有广阔的应用前景。然而，现阶段外周血循环标志物仍处于探索阶段，一些生物标志物如bTMB、循环PD-L1、免疫细胞亚型的检测需要特定的技术，而这些检测技术必须建立标准化的流程，各个标志物疗效预测的阈值仍需前瞻性的研究进一步确定。随着大数据和人工智能的发展，构建涵盖肿瘤特征及宿主免疫状态的多变量预测模型，将进一步提高预测效能并可以在治疗过程中从多个维度综合评估免疫治疗的效果，成为协助制定精准、个体化的治疗方案的有效工具，使越来越多的NSCLC患者从免疫治疗中获益。
